# Green Synthesis of Magnetite (Fe_3_O_4_) Nanoparticles Using Seaweed (*Kappaphycus alvarezii*) Extract

**DOI:** 10.1186/s11671-016-1498-2

**Published:** 2016-06-02

**Authors:** Yen Pin Yew, Kamyar Shameli, Mikio Miyake, Noriyuki Kuwano, Nurul Bahiyah Bt Ahmad Khairudin, Shaza Eva Bt Mohamad, Kar Xin Lee

**Affiliations:** Malaysia–Japan International Institute of Technology, Universiti Teknologi Malaysia, Jalan Sultan Yahya Petra, 54100 Kuala Lumpur, Malaysia

**Keywords:** Green synthesis, Fe_3_O_4_ nanoparticles, Seaweed, *Kappaphycus alvarezii*, Transmission electron microscopy

## Abstract

In this study, a simple, rapid, and eco-friendly green method was introduced to synthesize magnetite nanoparticles (Fe_3_O_4_-NPs) successfully. Seaweed *Kappaphycus alvarezii* (*K. alvarezii*) was employed as a green reducing and stabilizing agents. The synthesized Fe_3_O_4_-NPs were characterized with X-ray diffraction (XRD), ultraviolet-visible spectroscopy (UV-Vis), Fourier transform infrared (FT-IR), and transmission electron microscopy (TEM) techniques. The X-ray diffraction planes at (220), (311), (400), (422), (511), (440), and (533) were corresponding to the standard Fe_3_O_4_ patterns, which showed the high purity and crystallinity of Fe_3_O_4_-NPs had been synthesized. Based on FT-IR analysis, two characteristic absorption peaks were observed at 556 and 423 cm^−1^, which proved the existence of Fe_3_O_4_ in the prepared nanoparticles. TEM image displayed the synthesized Fe_3_O_4_-NPs were mostly in spherical shape with an average size of 14.7 nm.

## Background

With the recent rapid development and evolvement of technology, human beings have put their faith in nanotechnology and believe that it can ameliorate their current living standard [1]. As a consequence, the nanoparticle has drawn a huge interest from researchers globally due to specific characteristics such as shape, size, and distribution, which could be utilized in a distinct field of applications [2]. Synthesis of Fe_3_O_4_-NPs has been carried out because of its unique properties, such as being superparamagnetic [3], biocompatible, biodegradable, and expected to be non-toxic to humans [4–6]. These unique properties allow Fe_3_O_4_-NPs to be widely used in different areas of applications, such as catalysis [7, 8], magnetic storage media [9], biosensors [10], magnetic resonance imaging (MRI) [11, 12], and targeted drug delivery [13–15].

Numerous methods of fabrication of Fe_3_O_4_-NPs can be employed, such as sol-gel method [16], solid state synthesis [17], and flame spray synthesis [18]. In contrast to the time-consuming chemical and physical methods which involve complicated procedures, green method is much easier and safer to use, and plant-mediated synthesis of nanoparticles is still a new scheme and the outcome is yet to be studied. There are a couple of successful studies in synthesizing Fe_3_O_4_-NPs by using plant extract. For instance, fruit extract of *Artemisia annua* [19], leaf extract of *Perilla frutescens* [20], *Tridax procumbens* [21] and *caricaya papaya* [22], peel extract of plantain [23], and also seed extract of grape *proanthocyanidin* [24]. However, there are only finite studies on the synthesis of Fe_3_O_4_-NPs from marine plants.

*Kappaphycus alvarezii* (*K. alvarezii*) is a type of red seaweed from the family of *Solieriaceae*. It is well-known in the food industries for its gelling properties [25]. Carrageenan gives the thickening characteristic, which can be used as a function of green stabilizer in synthesis of nanoparticles without using hazardous chemicals. Based on the literature review, there are still no specific researches done on seaweed *K. alvarezii* for the Fe_3_O_4_-NPs synthesis, and this inspires and motivates us to work on this. Hence, in this research, a novel green method of synthesizing Fe_3_O_4_-NPs using seaweed *K. alvarezii* is proposed.

## Main Text

### Methods

#### Materials

Iron (II) chloride tetrahydrate (FeCl_2_.4H_2_O ≥ 99 %) and iron (III) chloride hexahydrate (FeCl_3_. 6H_2_O, 97 %) were purchased from Sigma-Aldrich. Sodium hydroxide (NaOH) was obtained from R&M Chemicals. All the chemicals were used without further purification. The seaweed *K. alvarezii* is a type of red seaweed which was acquired from Sabah, Malaysia. All the aqueous solutions were prepared by deionized water from ELGA Lab Water Purification System, UK. The sensION+ MM374 GLP 2 channel Benchtop Meter was employed to control the pH of the solution. An Esco Isotherm Forced Convection Laboratory Oven was used to dry the washed sample.

#### Preparation of *Kappaphycus alvarezii* Extract

The seaweed was washed under running water to remove dirt, salt, and foreign particles. Then, it was soaked overnight (24 h) in deionized water to bleach the yellowish color so that it became colorless. After that, the seaweed was rinsed and dried under sunlight for 3 days. The dried seaweed was chopped into small pieces before being blended using a hammer mill with a 3-mm filter diameter. Finally, the dried seaweed was stored until further processing. The dried seaweed reduced the storage space required and can be stored for a number of years without appreciable loss of the gelling property. In this study, 0.5 g of dried seaweed was weighed and soaked in 50 ml of deionized water for 24 h. The resulting extract was used as a seaweed extract solution.

#### Synthesis of *K. alvarezii*/Fe_3_O_4_-NPs

For the synthesis of *K. alvarezii*/Fe_3_O_4_-NPs, firstly, a solution of Fe^3+^ and Fe^2+^ with a 2:1 M ratio was added into the seaweed extract to obtain a yellowish colloidal solution. Then, the freshly prepared 1.0 M of NaOH was added drop-wise to the solution under continuous stirring. The pH of the solution was adjusted to pH 11. The solution was then stirred for 1 h to homogenize the solution and also for the completion of reaction. After that, the as-synthesized Fe_3_O_4_-NPs were separated by using a permanent magnet. The Fe_3_O_4_-NPs were washed for several times by using deionized water. The nanoparticles were dried in an oven at around 70 °C for 24 h. The dried sample was stored in an air-tight container for further characterization. All the experiments were conducted at ambient temperature.

#### Characterization of *K. alvarezii*/Fe_3_O_4_-NPs

The presence and phase purity of the synthesized *K. alvarezii*/Fe_3_O_4_-NPs were examined by using a PANalytical X’Pert PRO X-ray diffractometer (XRD). The dried sample was performed at an applied current of 20 mA and accelerating voltage of 45 kV with Cu Kα radiation (*λ* = 1.54 Å) at 2θ angle configuration scanning from 5° to 80° (scanning rate = 2θ/min). The UV-Vis spectrum of Fe_3_O_4_-NPs was determined using Shimadzu UV-Visible Spectrophotometer (UV-1800). Fourier transform infrared (FT-IR) spectroscopy was used to study the presence of the biomolecules which are responsible for the synthesis of Fe_3_O_4_-NPs. Dried samples were ground with potassium bromide (KBr) to produce pellet, which was examined in a wavelength range of 400–4000 cm^−1^. The infrared absorption peaks were obtained from Thermo Scientific Nicolet 6700 Spectrometer. The size and morphology of the synthesized Fe_3_O_4_-NPs were observed using FEI TECNAI G2 F20 transmission electron microscope (TEM). The microscope had accelerating voltage from 20 to 200 kV and standard magnification from 22 X to 930 KX. The aqueous dispersion of the nanoparticles was dropped on 300-mesh copper grids and air-dried before viewing under a microscope. TEM images were acquired using a SC1000 ORIUS CCD Camera.

## Discussion

**Fig. 1:**
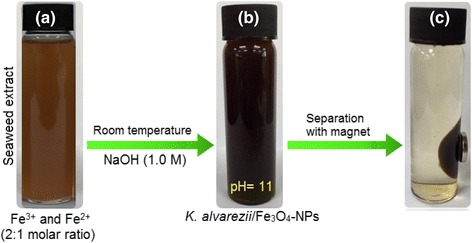
**a** Solution of Fe^3+^ and Fe^2+^ with 2:1 M ratio and seaweed *K. alvarezii* extract. **b** *K. alvarezii*/Fe_3_O_4_-NPs. **c** Separation of synthesized Fe_3_O_4_-NPs from reaction mixture using an external magnet

After addition of NaOH and stirring the solution for 1 h, the color of the reaction mixture of iron chloride salts and seaweed extract changed from light brown (Fig. 1a) to black (Fig. 1b), which indicates the formation of Fe_3_O_4_-NPs. Separation of Fe_3_O_4_-NPs could be done with the aid of an external permanent magnet. Figure 1c clearly reveals that the synthesized Fe_3_O_4_-NPs is able to be attracted by an external permanent magnet quickly, which proved that the nanoparticles possessed magnetic properties. Once the magnet was removed, the nanoparticles were dispersed readily by shaking.

Fe_3_O_4_ nanoparticles are prepared by adding a base to an aqueous mixture of Fe^3+^ and Fe^2+^ chloride at a 2:1 M ratio. The chemical reaction of Fe_3_O_4_ precipitation is given in Eqs. 1 and 2. The overall reaction may be written as follows:

1 $$ K.\  alvarezii + {\mathrm{H}}_2{\mathrm{O}}_{\left(\mathrm{l}\right)}+\mathrm{F}{\mathrm{e}}_{\left(\mathrm{a}\mathrm{q}\right)}^{2+} + \mathrm{F}{\mathrm{e}}_{\left(\mathrm{a}\mathrm{q}\right)}^{3+}\underrightarrow{Stirring}\kern.2em \left[K.\  alvarezii/\mathrm{F}{\mathrm{e}}^{3+}\hbox{-} \mathrm{F}{\mathrm{e}}^{2+}\right] $$

2 $$ \left[K.\  alvarezii/\mathrm{F}{\mathrm{e}}^{3+}\hbox{-} \mathrm{F}{\mathrm{e}}^{2+}\right] + 80{\mathrm{H}}_{\left(\mathrm{a}\mathrm{q}\right)}^{-}\underrightarrow{Stirring}\kern.2em \left[K.\  alvarezii/F{e}_3{O}_4\right]{\downarrow}_{(s)} + 4{\mathrm{H}}_2{\mathrm{O}}_{\left(\mathrm{a}\mathrm{q}\right)} $$

The precipitation occurs because of the Fe_3_O_4_-NPs have a high tendency to aggregate into agglomerates as to decrease the energy associated with the large surface area to volume ratio, a phenomenon which is likely deteriorated by the low surface charge [26].

### UV-Vis Spectral Analysis

**Fig. 2:**
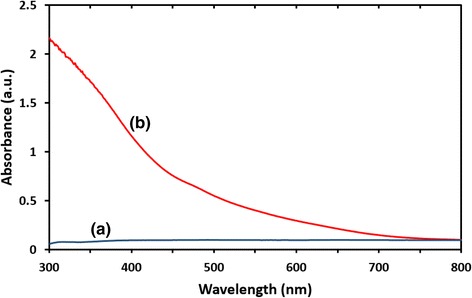
UV-vis absorption spectra of (***a***) seaweed *K. alvarezii.* (***b***) Synthesized Fe_3_O_4_-NPs

The UV-Vis spectroscopy of seaweed *K. alvarezii* and synthesized Fe_3_O_4_-NPs are shown in Fig. 2. Seaweed *K. alvarezii* does not show any absorption peak (blue). However, the synthesized Fe_3_O_4_-NPs reveals continuous absorption in the visible range of 300–800 nm without any strong absorption peak (red). Studies from Basavegowda et al. had reported identical UV-Vis spectra for Fe_3_O_4_-NPs synthesized using *A. annua* and *P. frutescens* extracts [19, 20].

### XRD Analysis

**Fig. 3:**
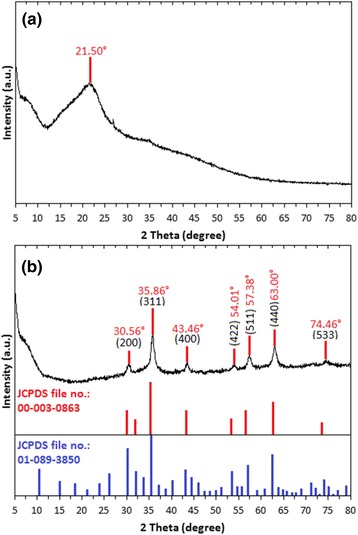
XRD patterns of **a** seaweed *K. alvarezii.* **b** Synthesized Fe_3_O_4_-NPs

Phase purity and crystallinity of the synthesized Fe_3_O_4_-NPs can be identified via XRD analysis. The XRD patterns of seaweed *K. alvarezii* and synthesized Fe_3_O_4_-NPs are shown in Fig. 3. A broad diffraction peak was observed (Fig. 3a) at 21.50°, which is attributed to seaweed *K. alvarezii*. This is confirmed by a study which showed unanimous XRD patterns of seaweed *K. alvarezii* that were used in the synthesis of Cu@Cu_2_O core shell nanoparticles [27]. The diffraction peaks of synthesized Fe_3_O_4_-NPs (Fig. 3b) were detected at 2θ = 30.56°, 35.86°, 43.46°, 54.01°, 57.38°, 63.00°, and 74.46°, which are assigned to the crystal planes of (200), (311), (400), (422), (511), (440), and (533), respectively. The analyzed diffraction peaks were matched well with the standard magnetite XRD patterns with JCPDS file no: 00-003-0863, which declared the crystallographic system of cubic structure. Besides, the synthesized nanoparticles were affirmed to be Fe_3_O_4_ but not maghemite (γ-Fe_2_O_3_) by comparing the XRD patterns with the standard maghemite JCPDS file no.: 01-089-3850. A huge difference can be clearly seen in that the XRD patterns of γ-Fe_2_O_3_ consist of many peaks, unlike Fe_3_O_4_ which only involves few peaks. Estimation of the crystallite size of synthesized Fe_3_O_4_-NPs can be calculated by using the Debye-Scherrer equation [28], which reveals a relationship between X-ray diffraction peak broadening and crystallite size. The Debye-Scherrer equation is shown as follows:

$$ d=\frac{k\lambda }{\beta_{hkl} cos{\theta}_{hkl}}, $$

where *d* is the crystallite size of synthesized Fe_3_O_4_-NPs for (*hkl*) phase, *k* is Scherrer constant (0.9), *λ* is the X-ray wavelength of radiation for Cu Kα (0.154 nm), β_hkl_ is the full-width at half maximum (FWHM) at (*hkl*) peak in radian, and *θ*_hkl_ is the diffraction angle for (*hkl*) phase. Using the equation, the estimated crystallite size of synthesized Fe_3_O_4_-NPs was 16.79 nm, which was calculated from the full-width at half maximum of the Fe_3_O_4_ (311) diffraction peak [29] at 2θ = 35.86°. Based on the X-ray diffraction pattern, the synthesized Fe_3_O_4_-NPs were figured out to be high purity crystalline, as no impurity peak was observed.

### Transmission Electron Microscopy Study

**Fig. 4:**
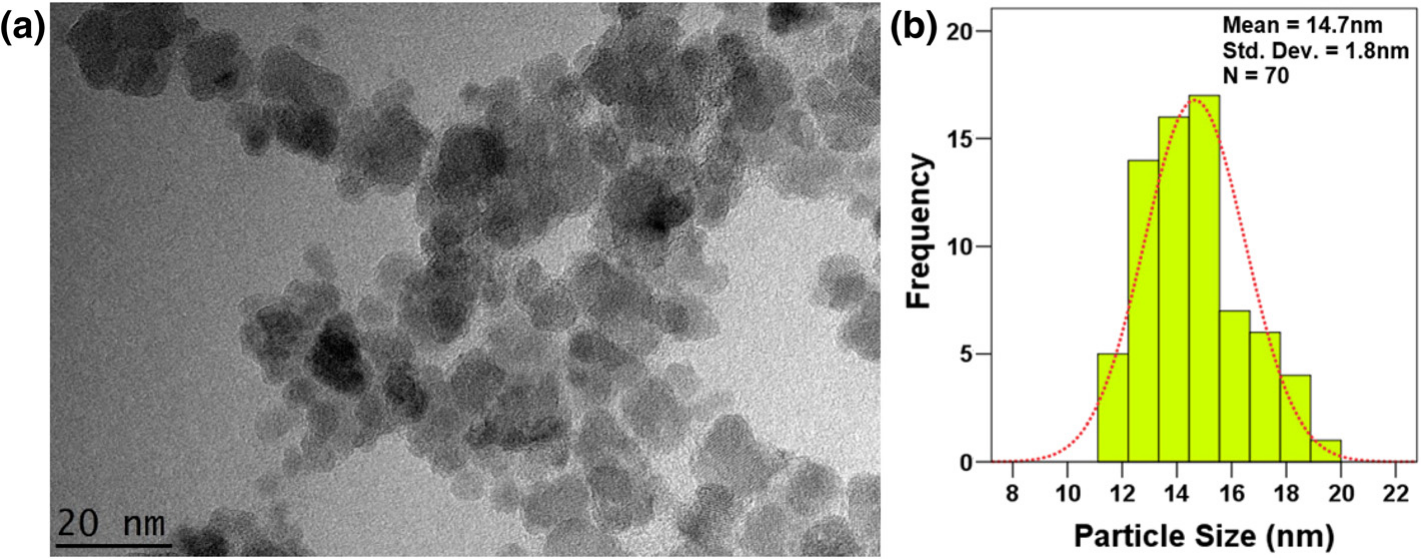
**a** TEM image of the synthesized Fe_3_O_4_-NPs at magnification of 35 KX. **b** Size distribution histogram of the synthesized Fe_3_O_4_-NPs

The size and morphology of the synthesized Fe_3_O_4_-NPs were analyzed by using TEM. The TEM image of synthesized Fe_3_O_4_-NPs (Fig. 4a) showed that majority of the nanoparticles were in spherical shape. The image revealed that most of the particles were agglomerated, which might be due to the thickening properties of seaweed *K. alvarezii* or the presence of hydroxyl groups from the extract [30]. Besides, the tendency of agglomeration is not surprising as the synthesized Fe_3_O_4_-NPs is small in size and possess magnetic characteristics [31]. A histogram of particle size distribution was drawn according to the size of 70 nanoparticles (Fig. 4b). The mean particle size was 14.7 nm with the standard deviation of 1.8 nm. The crystallite size of the synthesized Fe_3_O_4_-NPs was found to be 16.7 nm from XRD analysis, which is in an agreement with the result obtained from the TEM that shows a size distribution between 11.0 and 20.0 nm. A few very small spherical objects can be observed from the image which might be due to the residue of seaweed *K. alvarezii.*

#### Fourier Transform Infrared Study

**Fig. 5:**
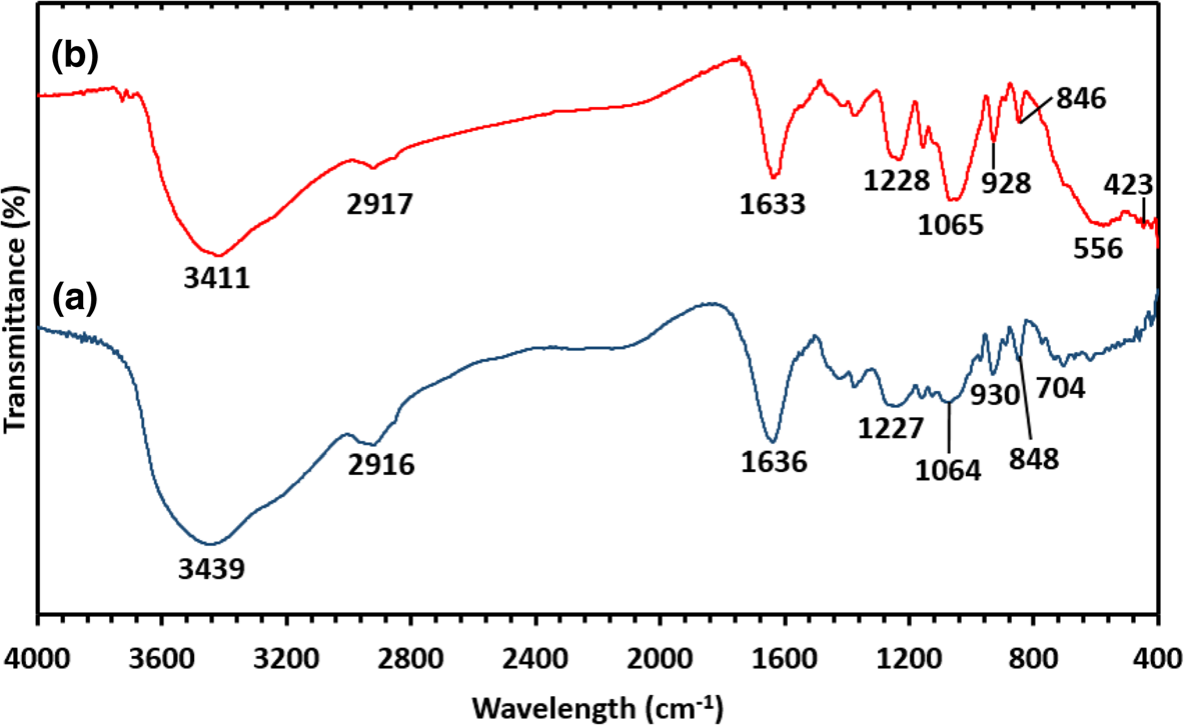
FT-IR spectra of (***a***) seaweed *K. alvarezii.* (***b***) Synthesized Fe_3_O_4_-NPs

FT-IR spectroscopy was performed to determine the functional groups of seaweed *K. alvarezii* that acted as a stabilizer and capping agent in the synthesis of Fe_3_O_4_–NPs. The spectra of the seaweed *K. alvarezii* extract revealed strong absorption bands at 3439, 2916, 1636, 1227, 1064, 930, 848, and 704 cm^−1^ (Fig. 5a), while the absorption bands of synthesized Fe_3_O_4_-NPs were observed at 3411, 2917, 1633, 1228, 1065, 928, 846, 556, and 423 cm^−1^ (Fig. 5b). The absorption peak of 3439 cm^−1^ in the *K. alvarezii* extract indicated the O–H stretching vibration. The absorption peaks at 2916 cm^−1^ contributed to the C–H stretching vibrations of the –CH_2_ functional group [32], while 1636 cm^−1^ were attributed to the C–H bending overtone band of aromatic compound. Absorption peak at 1227 cm^−1^ corresponded to the asymmetric stretching vibration of the sulfate group [33], whereas peak at 1064 cm^−1^ was assigned to the C–O stretching band related to the C–O–SO_3_ group [34]. The absorption peaks 930, 848, and 704 cm^−1^ revealed the existence of aromatic C–H bending band. All the bands were shifted, indicating the participation and interaction of nanoparticles with the seaweed *K. alvarezii* extract [35, 36]. Two significant new peaks were found at 556 and 423 cm^−1^ in the spectra of synthesized Fe_3_O_4_-NPs, which is associated with the stretching vibration mode of Fe–O. The metal-oxygen band at 556 cm^−1^ corresponded to intrinsic stretching vibrations of metal at the tetrahedral site, while the metal-oxygen band found at 423 cm^−1^ was assigned to octahedral-metal stretching of Fe–O [37]. The formation of Fe_3_O_4_-NPs was confirmed with these characteristic peaks as the peaks laying in the region between 400 and 600 cm^−1^ were corresponding to Fe_3_O_4_ [28].

**Fig. 6:**
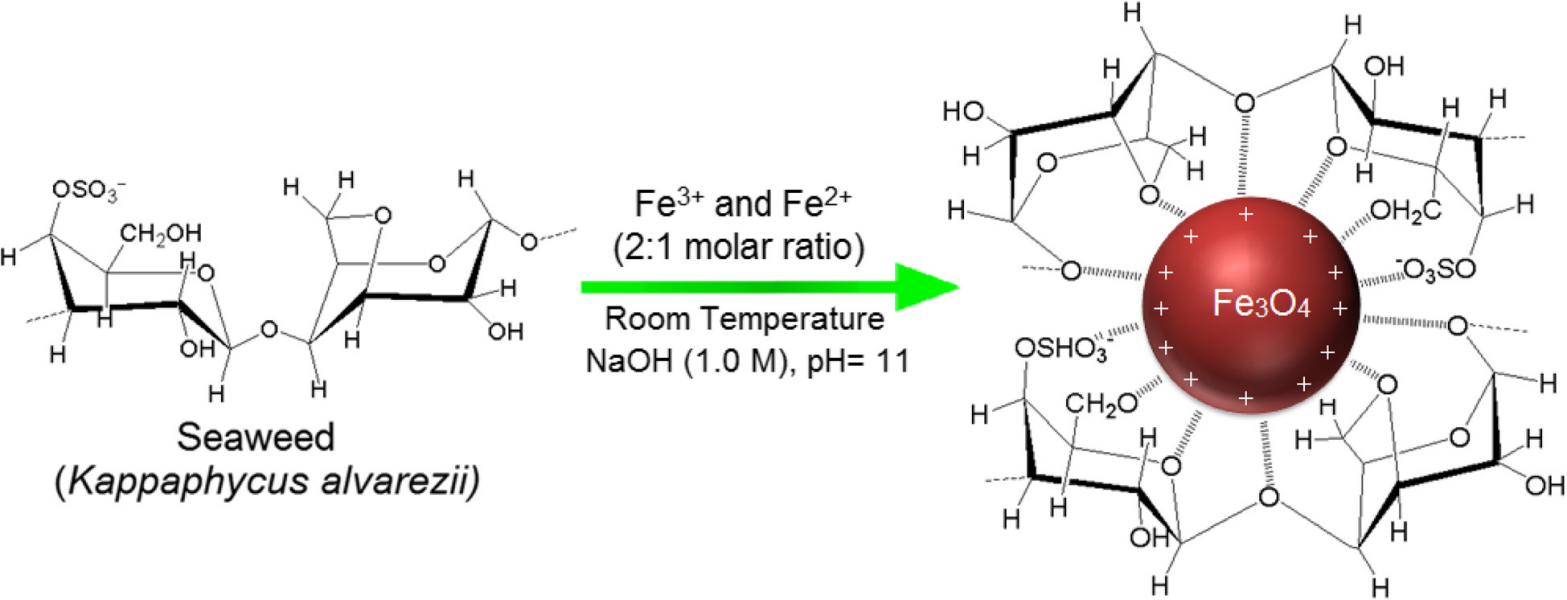
Schematic diagram demostrating the interaction between Fe_3_O_4_-NPs-charged groups which are capped by seaweed *K. alvarezii*

A presumed schematic diagram of formation of *K. alvarezii*/Fe_3_O_4_-NPs is illustrated in Fig. 6, where the presence of van der Waals forces holds the positively charged Fe_3_O_4_-NPs and negatively charged groups presenting in the molecular structure of seaweed *K. alvarezii* together [38].

## Conclusions

In this study, Fe_3_O_4_-NPs were synthesized successfully by a simple and green approach using the seaweed *K. alvarezii* extract without utilizing any chemical-reducing agent and stabilizer. Based on the XRD analysis studied, a high purity crystalline of Fe_3_O_4_-NPs was prepared. FT-IR spectroscopy showed the involvement biomolecules present in the extract of seaweed *K. alvarezii*, which were verified in the synthesizing process of Fe_3_O_4_-NPs. The formation of Fe_3_O_4_-NPs was confirmed due to the noticeable absorption peaks at 556 and 423 cm^−1^. TEM result revealed the size and morphology of the synthesized Fe_3_O_4_-NPs. Most of the particles possessed spherical shapes with average particle sizes of 14.7 nm. The non-toxic green synthesized Fe_3_O_4_-NPs are expected suitable to be employed in various fields of applications, especially in biomedical applications.

## Abbreviations

*NPs* nanoparticles, *K. alvarezii Kappaphycus alvarezii*, *XRD* X-ray diffraction, *UV-Vis* ultraviolet-visible spectroscopy, *FT-IR* Fourier transform infrared, TEM transmission electron microscopy
